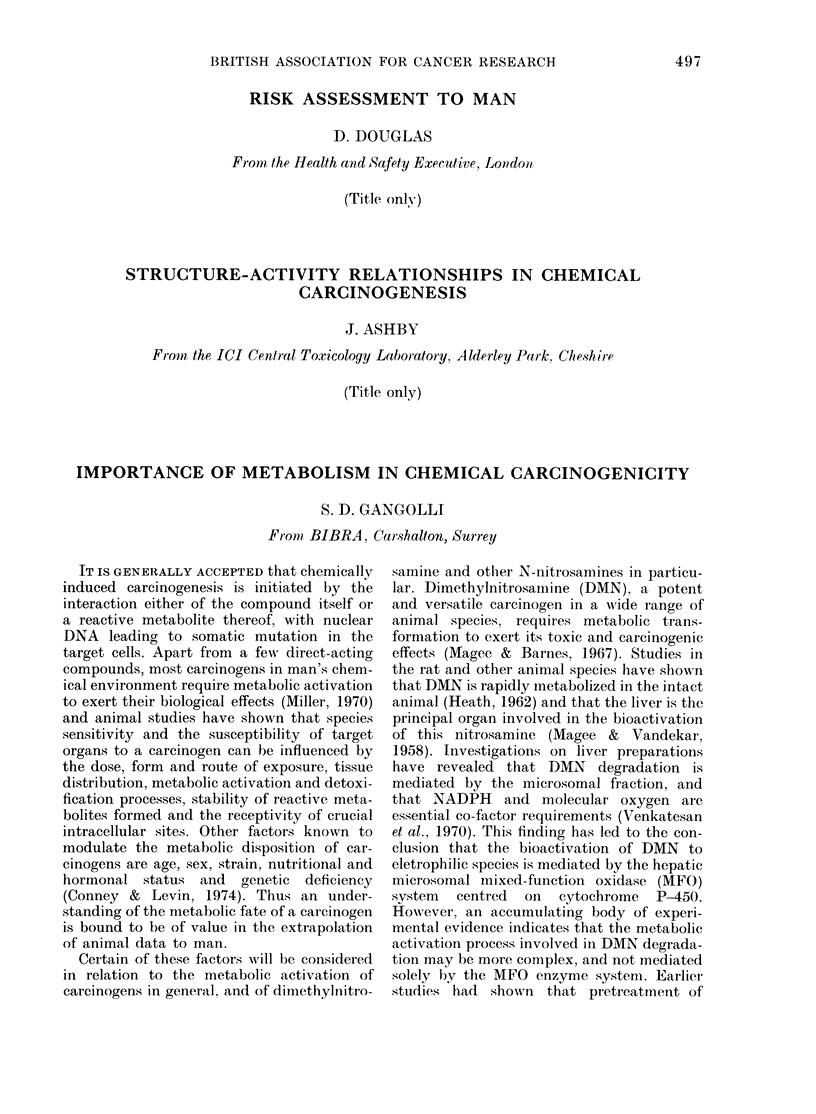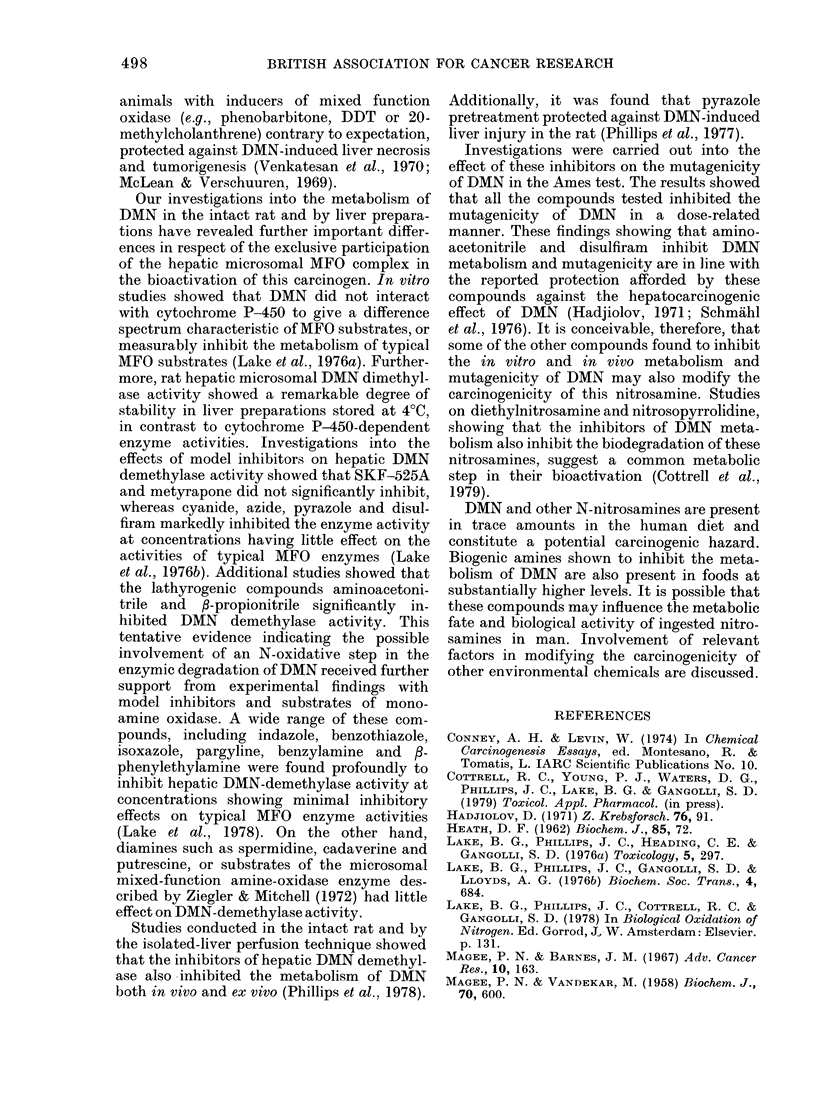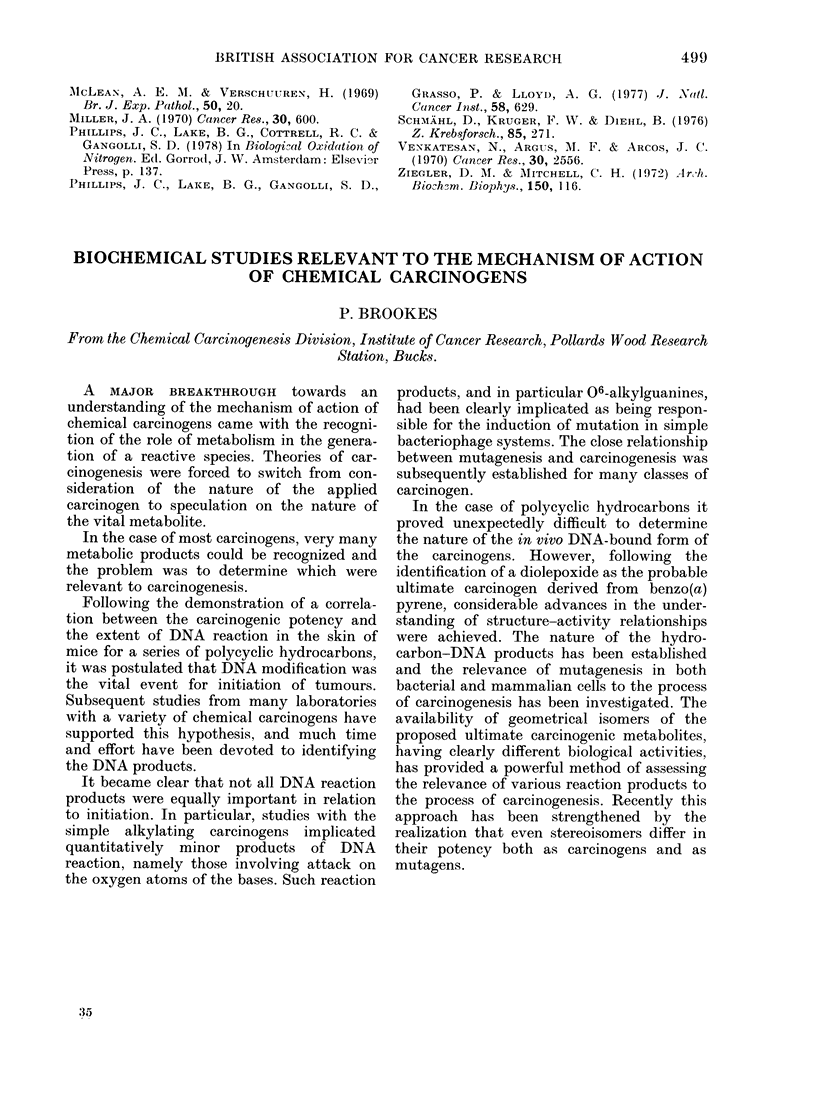# Importance of metabolism in chemical carcinogenicity.

**DOI:** 10.1038/bjc.1980.79

**Published:** 1980-03

**Authors:** S. D. Gangolli


### RISK ASSESSMENT TO MAN

D. DOUGLAS

*From the Health and Safety Executive, London*

(Title only)

### STRUCTURE-ACTIVITY RELATIONSHIPS IN CHEMICAL CARCINOGENESIS

J. ASHBY

*From the ICI Central Toxicology Laboratory, Alderley Park, Cheshire*

(Title only)

### IMPORTANCE OF METABOLISM IN CHEMICAL CARCINOGENICITY

S. D. GANGOLLI

*From BIBRA, Carshalton, Surrey*

IT IS GENERALLY ACCEPTED that chemically induced carcinogenesis is initiated by the interaction either of the compound itself or a reactive metabolite thereof, with nuclear DNA leading to somatic mutation in the target cells. Apart from a few direct-acting compounds, most carcinogens in man's chemical environment require metabolic activation to exert their biological effects (Miller, 1970) and animal studies have shown that species sensitivity and the susceptibility of target organs to a carcinogen can be influenced by the dose, form and route of exposure, tissue distribution, metabolic activation and detoxification processes, stability of reactive metabolites formed and the receptivity of crucial intracellular sites. Other factors known to modulate the metabolic disposition of carcinogens are age, sex, strain, nutritional and hormonal status and genetic deficiency (Conney & Levin, 1974). Thus an understanding of the metabolic fate of a carcinogen is bound to be of value in the extrapolation of animal data to man.

Certain of these factors will be considered in relation to the metabolic activation of carcinogens in general, and of dimethylnitrosamine and other N-nitrosamines in particular. Dimethylnitrosamine (DMN), a potent and versatile carcinogen in a wide range of animal species, requires metabolic transformation to exert its toxic and carcinogenic effects (Magee & Barnes, 1967). Studies in the rat and other animal species have shown that DMN is rapidly metabolized in the intact animal (Heath, 1962) and that the liver is the principal organ involved in the bioactivation of this nitrosamine (Magee & Vandekar, 1958). Investigations on liver preparations have revealed that DMN degradation is mediated by the microsomal fraction, and that NADPH and molecular oxygen are essential co-factor requirements (Venkatesan *et al.*, 1970). This finding has led to the conclusion that the bioactivation of DMN to eletrophilic species is mediated by the hepatic microsomal mixed-function oxidase (MFO) system centred on cytochrome P–450. However, an accumulating body of experimental evidence indicates that the metabolic activation process involved in DMN degradation may be more complex, and not mediated solely by the MFO enzyme system. Earlier studies had shown that pretreatment of animals with inducers of mixed function oxidase (*e.g.*, phenobarbitone, DDT or 20-methylcholanthrene) contrary to expectation, protected against DMN-induced liver necrosis and tumorigenesis (Venkatesan *et al.*, 1970; McLean & Verschuuren, 1969).

Our investigations into the metabolism of DMN in the intact rat and by liver preparations have revealed further important differences in respect of the exclusive participation of the hepatic microsomal MFO complex in the bioactivation of this carcinogen. *In vitro* studies showed that DMN did not interact with cytochrome P–450 to give a difference spectrum characteristic of MFO substrates, or measurably inhibit the metabolism of typical MFO substrates (Lake *et al.*, 1976*a*). Furthermore, rat hepatic microsomal DMN dimethylase activity showed a remarkable degree of stability in liver preparations stored at 4°C, in contrast to cytochrome P–450-dependent enzyme activities. Investigations into the effects of model inhibitors on hepatic DMN demethylase activity showed that SKF–525A and metyrapone did not significantly inhibit, whereas cyanide, azide, pyrazole and disulfiram markedly inhibited the enzyme activity at concentrations having little effect on the activities of typical MFO enzymes (Lake *et al.*, 1976*b*). Additional studies showed that the lathyrogenic compounds aminoacetonitrile and β-propionitrile significantly inhibited DMN demethylase activity. This tentative evidence indicating the possible involvement of an N-oxidative step in the enzymic degradation of DMN received further support from experimental findings with model inhibitors and substrates of monoamine oxidase. A wide range of these compounds, including indazole, benzothiazole, isoxazole, pargyline, benzylamine and β-phenylethylamine were found profoundly to inhibit hepatic DMN-demethylase activity at concentrations showing minimal inhibitory effects on typical MFO enzyme activities (Lake *et al.*, 1978). On the other hand, diamines such as spermidine, cadaverine and putrescine, or substrates of the microsomal mixed-function amine-oxidase enzyme described by Ziegler & Mitchell (1972) had little effect on DMN-demethylase activity.

Studies conducted in the intact rat and by the isolated-liver perfusion technique showed that the inhibitors of hepatic DMN demethylase also inhibited the metabolism of DMN both *in vivo* and *ex vivo* (Phillips *et al.*, 1978). Additionally, it was found that pyrazole pretreatment protected against DMN-induced liver injury in the rat (Phillips *et al.*, 1977).

Investigations were carried out into the effect of these inhibitors on the mutagenicity of DMN in the Ames test. The results showed that all the compounds tested inhibited the mutagenicity of DMN in a dose-related manner. These findings showing that aminoacetonitrile and disulfiram inhibit DMN metabolism and mutagenicity are in line with the reported protection afforded by these compounds against the hepatocarcinogenic effect of DMN (Hadjiolov, 1971; Schmähl *et al.*, 1976). It is conceivable, therefore, that some of the other compounds found to inhibit the *in vitro* and *in vivo* metabolism and mutagenicity of DMN may also modify the carcinogenicity of this nitrosamine. Studies on diethylnitrosamine and nitrosopyrrolidine, showing that the inhibitors of DMN metabolism also inhibit the biodegradation of these nitrosamines, suggest a common metabolic step in their bioactivation (Cottrell *et al.*, 1979).

DMN and other N-nitrosamines are present in trace amounts in the human diet and constitute a potential carcinogenic hazard. Biogenic amines shown to inhibit the metabolism of DMN are also present in foods at substantially higher levels. It is possible that these compounds may influence the metabolic fate and biological activity of ingested nitrosamines in man. Involvement of relevant factors in modifying the carcinogenicity of other environmental chemicals are discussed.

REFERENCES

Conney, A. H. & Levin, W. (1974) In *Chemical Carcinogenesis Essays*, ed. Montesano, R. & Tomatis, L. IARC Scientific Publications No. 10.

Cottrell, R. C., Young, P. J., Waters, D. G., Phillips, J. C., Lake, B. G. & Gangolli, S. D. (1979) *Toxicol. Appl. Pharmacol.* (in press).

Hadjiolov, D. (1971) *Z. Krebsforsch.* **76**, 91.

Heath, D. F. (1962) *Biochem. J.*, **85**, 72.

Lake, B. G., Phillips, J. C., Heading, C. E. & Gangolli, S. D. (1976*a*) *Toxicology*, **5**, 297.

Lake, B. G., Phillips, J. C., Gangolli, S. D. & Lloyds, A. G. (1976*b*) *Biochem. Soc. Trans.*, **4**, 684.

Lake, B. G., Phillips, J. C., Cottrell, R. C. & Gangolli, S. D. (1978) In *Biological Oxidation of Nitrogen*. Ed. Gorrod, J. W. Amsterdam: Elsevier. p. 131.

Magee, P. N. & Barnes, J. M. (1967) *Adv. Cancer Res.*, **10**, 163.

Magee, P. N. & Vandekar, M. (1958) *Biochem. J.*, **70**, 600.

McLEAN, A. E. M. & VERSCHUUREN, H. (1969) *Br. J. Exp. Pathol.*, **50**, 20.
MILLER, J. A. (1970) *Cancer Res.*, **30**, 600.
PHILLIPS, J. C., LAKE, B. G., COTTRELL, R. C. & GANGOLLI, S. D. (1978) In *Biological Oxidation of Nitrogen.* Ed. Gorrod, J. W. Amsterdam: Elsevier Press, p. 137.
PHILLIPS, J. C., LAKE, B. G., GANGOLLI, S. D., GRASSO, P. & LLOYD, A. G. (1977) *J. Natl. Cancer Inst.*, **58**, 629.
SCHMÄHL, D., KRUGER, F. W. & DIEHL, B. (1976) *Z. Krebsforsch.*, **85**, 271.
VENKATESAN, N., ARGUS, M. F. & ARCOS, J. C. (1970) *Cancer Res.*, **30**, 2556.
ZIEGLER, D. M. & MITCHELL, C. H. (1972) *Arch. Biochem. Biophys.*, **150**, 116.